# A novel transcatheter tricuspid annuloplasty for severe tricuspid valve regurgitation via the K-clip™ system: early experience in China

**DOI:** 10.3389/fcvm.2025.1598644

**Published:** 2025-10-01

**Authors:** Rongfeng Xu, Lijuan Chen, Xiaoli Zhang, Xiuxia Ding, Zhen Wang, Qitong Lu, Xiaoguo Zhang, Jiandong Ding, Genshan Ma

**Affiliations:** ^1^ Department of Cardiology, Zhongda Hospital, Southeast University, Nanjing, China; ^2^ Department of Anesthesiology, Zhongda Hospital, Southeast University, Nanjing, China; ^3^ Department of Cardio-Thoracic Surgery, Zhongda Hospital, Southeast University, Nanjing, China

**Keywords:** tricuspid regurgitation, tricuspid annuloplasty, transcatheter tricuspid valve interventions, K-Clip, transcatheter tricuspid repair

## Abstract

**Background:**

Patients who suffer from severe tricuspid regurgitation (TR) do not undergo standard care therapy because of the high surgical risk. As a result, safer and less invasive techniques are being sought after internationally. The objective of this study was to investigate the feasibility and safety of the K-Clip™ device, a novel interventional tricuspid annuloplasty system designed for transcatheter tricuspid repair that is positioned using ultrasound technology and fluoroscopy.

**Methods:**

Four patients with severe symptomatic TR (3 with massive and 1 with torrential TR) and high surgical risk [STS score of 6.7 (5.6–11.1)] underwent tricuspid annular repair with the K-Clip™ device guided by echocardiography and fluoroscopy. Echocardiographic measurements [vena contracta width, regurgitant volume, effective regurgitant orifice area (EROA)], quality-of-life (QoL) measurements [NYHA functional class, Kansas City Cardiomyopathy Questionnaire score (KCCQ), and the 6-min walk test (6MWT)] were performed before the procedure and at the 30-day follow-up assessment.

**Results:**

The K-Clip™ device was successfully implanted in all four patients (2 patients with 2 clips each and 2 patients with 1 clip each). No procedural or 30-day major adverse events occurred. The TR was reduced by at least 1 grade in all patients. EROA (0.93 ± 0.40 mm^2^ VS 0.42 ± 0.11 mm^2^, *p* < 0.05), vena contracta width (17.95 ± 8.19 mm VS 7.48 ± 1.87 mm, *p* < 0.05) and regurgitant volume (97.00 ± 46.41 ml VS 43.50 ± 17.13 ml, *p* < 0.05) were obviously reduced at 30 days after the procedure. Significant improvements in the NYHA functional class, KCCQ score (37.58 ± 6.48 VS 58.55 ± 5.13, *p* < 0.01), and 6MWT (239.67 ± 31.64 m VS 402.67 ± 41.53 m, *p* < 0.05) outcome were observed at the 30-day follow-up visit.

**Conclusions:**

This report on the early experience of transcatheter tricuspid repair with the K-Clip™ in China revealed notable clinical improvement, acceptable safety, and high procedural success. Larger prospective trials with extended follow-up periods are required to validate these encouraging preliminary findings and to clarify the effects of the K-Clip™ on clinical outcomes.

## 1 Introduction

Due to either pressure or volume overload, the right ventricle (RV) remodels, resulting in leaflet tethering and annular dilation, which in turn causes functional tricuspid valve regurgitation (FTR) (1). Chronic severe TR causes the RV to overstress its walls and experience volume overload, which exacerbates TR and causes harmful remodeling. TR is a prevalent valvular disease. In the US, 1.6 million people suffer from FTR (2). The prognosis and prevalence of TR in China are still poorly understood. Studies on the incidence and results of TR in China have been conducted, in which 1,870 (1.39%) patients had severe TR (3+/4+), 3,987 (2.96%) patients had mild TR (+), and 3,015 (2.22%) patients had moderate TR (2+) (3). FTR, however, happens in a sizable fraction of individuals following successful therapy of primary left-valve disease. The development of isolated TR is connected with several key variables, including the presence of pacemaker leads and long-lasting atrial fibrillation (AF) in addition to severe TR within the years following left valve surgery (4). No matter how old or how well the heart works, severe TR has a 5-year survival rate of fewer than 50% and is linked to a bad prognosis (5). An important condition that is often seen, severe functional TR has been linked to both a significant rise in death rates and unsatisfactory therapeutic outcomes (6). Up to 8.8% of patients who have surgery to replace or repair their tricuspid valve die while they are in the hospital (7).

A promising option for treating individuals at high risk for open cardiac surgery is percutaneous technologies for secondary TR repair. Transcatheter tricuspid valve interventions (TTVIs) are therefore becoming more and more popular as a means of treating patients with TR who are at high risk of requiring open cardiac surgery (8, 9). In line with considerable clinical improvement, Zhang et al. recently reported that transcatheter tricuspid annuloplasty using the K-Clip™ technology displayed outstanding initial procedural success, acceptable safety, and an exceptional decrease in TR (10). During the course of the trial, no 30-day major adverse cardiac events (MACEs) were documented, and all 15 patients from the 3 centers got implants safely (10). The 30-day echocardiographic and clinical results of 39 patients treated with the K-Clip™ device for severe TR were also reported by Xu et al. (11). The 6-minute walk distance (6MWD) increased dramatically, the number of New York Heart Association (NYHA) class III-IV patients declined from 79.5% to 5.1%, and the Kansas City Cardiomyopathy Questionnaire score (KCCQ) clearly improved, according to their data, which also showed that TR severity was reduced by at least one grade in all patients. Additionally, we recently published the results of our center's first K-Clip™ tricuspid annuloplasty, which involved a patient with severe functional TR and a high surgical risk (12). Furthermore, we have collected some preliminary data and experiences as we have completed additional K-Clip™ cases in our center. Overall, the objective of our study was to show the feasibility of transcatheter tricuspid annuloplasty with the K-Clip™ (HuiHe Health care, Shanghai, China) in a single centre and its impact on the short-term clinical outcomes in 4 patients with severe TR. This report of our early experience notes that we successfully treated TR with a minimally invasive approach by clamping the expanded tricuspid annular tissue and anchoring parts of the device to reduce the area of the tricuspid valve orifice that could not be matched.

## 2 Methods

### 2.1 Study design and patients

This was a compassionate-use, prospective, single-center, single-arm study including 4 consecutive patients with symptomatic severe TR treated with K-Clip™ annuloplasty system between July 2021 and July 2022 in Zhongda hospital, Southeast university, Nanjing, China. Patients considered for this trial had to be older than 18 years of age, with associated significant (≥3+) TR and NYHA functional class ≥II under medication. Patients were assessed to be at high surgical risk [Society of Thoracic Surgeons (STS) mortality score ≥6.0] by the local heart team but with life expectancy longer than 1 year and left ventricular ejection fraction higher than 40%, tricuspid annular plane systolic excursion (TAPSE) ≥13 mm and systolic pulmonary artery pressure (sPAP) ≤55 mmHg. Exclusion criteria included among others, tricuspid stenosis, calcification of the tricuspid subvalvular apparatus or the chordae tendineae, intracardiac mass, history of endocarditis or active infection including endocarditis, and inability to perform transesophageal echocardiography (TEE). The patients and their family received a detailed explanation of the procedure, the desired outcome, and the risks. The procedure was approved by the local ethics committee of the Zhongda Hospital Southeast University for human subjects (2021ZDSYLL276-P01) and the patients provided informed consent for the procedure.

### 2.2 Procedural technique

The K-Clip ™ device for TR reduction was implanted under general anesthesia. As previously described, TEE and fluoroscopy were used to ensure correct implantation (13). Pre-procedural cardiac computed tomography (CT), transthoracic echocardiography (TTE) and TEE were conducted separately to evaluate the anatomical viability for K-Clip ™ device placement. Every echocardiogram image was examined at an independent core laboratory. The mid-posterior annulus was typically the target site for anchoring; however, when the anterior-lateral annulus-derived TR jet is dominant, the target point should be pushed forward to the anteroposterior commissure. The tricuspid annulus was measured using a cardiac CT scan in order to choose the optimal implant length and design the treatment, which reduced the chance of damage to the right coronary artery (RCA) and produced the images of interest. The tricuspid annulus length, as determined by a cardiac CT scan at the tricuspid valve's maximal diastolic opening, was the primary factor used to determine the device size.

**Figure 1:**
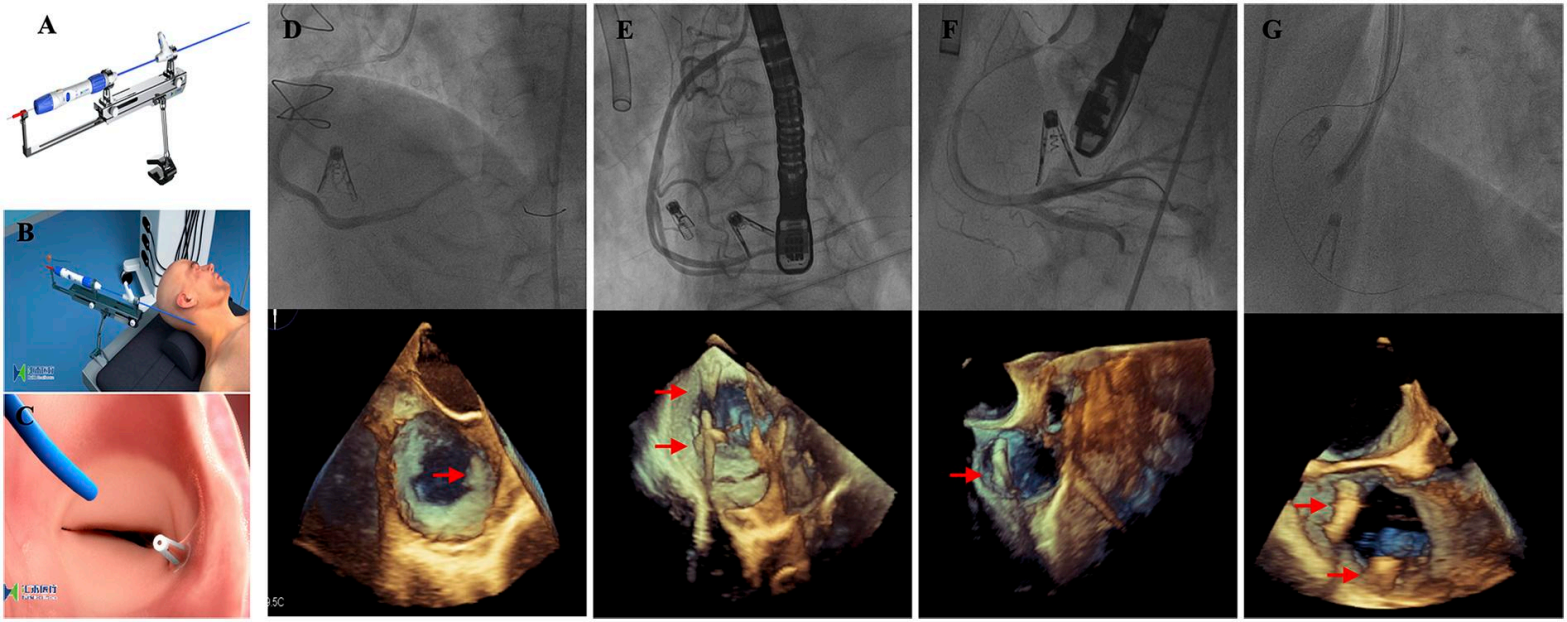
Procedural technique and results. **(A)** The K-Clip™ system; **(B)** The procedure was performed under general anesthesia and transjugular access; **(C)** After the clip released, the tricuspid annulus length was reduced; **(D–G)** The K-Clip™ device was successfully implanted in all four patients—2 patients with 2 clips each and 2 patients with 1 clip each, and 3D transesophageal echocardiography displayed the position of the clips (red arrow).

On the basis of echocardiography and CT measurements, a clip of K-Clip™ was selected for implantation. After signing informed consent, the procedure was carried out. The interventional procedure was performed under general anaesthesia, angiographic and TEE guidance (Figures 1A–C). Step 1; Trans-vascular access: After right jugular vein puncture, lead K-Clip ™ sheath introducer access RA throughout SVC, in addition, a guide catheter needs to be placed in the right coronary artery via femoral access because of the close proximity of the right coronary artery to the posterior portion of the tricuspid annulus; Step 2; Sheath Position: Figure out the tricuspid annulus guided by TEE and position the sheath introducer; Step 3; Anchor Deployment: Under the guidance of TTE and TEE, the anchor of K-Clip ™ reaches the midpoint of the posterior tricuspid annulus and tap the anchor screw into annulus; Step 4; Clamping Annulus Tissue: Under the guidance of TTE and TEE, clamp the tissue to fold the tricuspid annulus and then close the Clip arms slowly; Step 5; Sandwich Formed: Form the stable “Anchor-tissue-clamp arm” sandwich structure; Step 6; Release: Release the sandwich-like K-Clip ™ and withdraw the delivery system. Right jugular vein access was closed using a ProGlide closure system (Abbott Vascular) while manual pressure was held for haemostasis of the left femoral artery.

Following an RCA coronary angiography and echocardiographic confirmation of TR decrease, the final detachment should be carried out. During the process, intravenous heparin was given with the goal of achieving an active clotting time of 300–450 s. Following discharge, patients who did not meet the criteria for anticoagulation were typically prescribed dual-antiplatelet medication for a minimum of one month, or else it was advised to take an oral anticoagulant continuously.

### 2.3 Study endpoints

As previously mentioned, the procedural and clinical success constituted the main performance result (14, 15). The successful completion of the procedure was defined as: (a) the system was delivered and retrieved without occurrence; (b) at least one device was correctly implanted and deployed as planned at the time of the patient's departure from the cardiac catheterization laboratory, resulting in a decrease in post-procedural TR grade of ≥+1; and (c) the patient was discharged from the hospital without requiring surgery or percutaneous intervention. Following that, procedural success without any MACEs at 30 days was defined as clinical success. All-cause mortality, myocardial infarction, stroke, coronary artery perforation or stenosis, conduction disorders requiring a new permanent pacemaker implantation (PPM), major vascular complications, reintervention related to the device, arrhythmias, and recurrent heart failure were among the MACEs included in this study.

### 2.4 Statistical analysis

Categorical data were displayed as counts (percentages), while continuous variables were displayed as mean ± SD or as median (interquartile range) as appropriate. The Wilcoxon signed rank test was applied for categorical variables between baseline and follow-up, and paired student's *t*-tests were utilized to examine continuous variables. SPSS Statistics 27.0.0 was the program used for all data analysis. A two-sided *p*-value of less than 0.05 indicated the presence of a significant statistical difference.

## 3 Results

### 3.1 Baseline characteristics

The baseline characteristics of all enrolled patients are summarized in Table 1. A total of 4 patients with a mean age of 75.25 ± 5.12 years (2 male, 50.0%) were ultimately enrolled. All the patients were considered to be at high surgical risk, as indicated by a STS score of 6.7 (5.6–11.1) and TRI-SCORE of 6 (4–7). The comorbidities included hypertension (2 cases, 50.0%), diabetes mellitus (1 case, 25.0%), AF (4 cases, 100.0%), and stroke (1 case, 25.0%). Three patients (75.0%) had a NYHA functional class ≥III. In regard to the echocardiographic parameters, all patients had severe TR (including 3 with massive and 1 with torrential TR), with an average tricuspid sep-to-lateral annulus diameter of 43.14 ± 7.48 mm. The aetiology of TR was functional in all 4 patients.

**Table 1:** Patient characteristics.

Variables	*N* = 4
Age, years	75.25 ± 5.12
Sex, male (*n*, %)	2 (50.0)
STS score, %	6.7 (5.6–11.1)
TRI-SCORE	6 (4–7)
TRIVALVE score	3 (2.5–3.5)
Hypertension (*n*, %)	2 (50.0)
Diabetes (*n*, %)	1 (25.0)
AF (*n*, %)	4 (100.0)
Coronary heart disease (*n*, %)	1 (25.0)
PCI/CABG (*n*, %)	1 (25.0)
Stroke/TIA (*n*, %)	1 (25.0)
Peripheral artery disease (*n*, %)	0 (0.0)
Kidney dysfunction (*n*, %)	2 (50.0)
Anemia (*n*, %)	2 (50.0)
Prior valvular intervention (*n*, %)	0 (0.0)
Prior valvular surgery (*n*, %)	1 (25.0)
NYHA functional class (*n*, %)	
I/II	1 (25.0)
III	2 (50.0)
IV	1 (25.0)

STS score, society of thoracic surgeons score; AF, atrial fibrillation; PCI, percutaneous coronary intervention; CABG, coronary artery bypass graft; TIA, transient ischemic attack; NYHA, New York Heart Association.

### 3.2 Procedural results

The key procedural characteristics of our study population are summarized in Table 2. In all patients, 1 clip or 2 clips each were successfully applied and the delivery system was retrieved as intended (Figures 1D–G). Procedural success was achieved in 4/4 (100%) patients, and there was no conversion to surgery. The mean operation and fluoroscopy times were 93.6 ± 23.4 min and 30.4 ± 15.7 min, respectively. In general, TR severity was effectively reduced by at least 1 grade in all 4 patients (100.00%). One patient still exhibited severe TR after the procedure. No procedural-related complications were observed. No other major adverse cardiac or cerebrovascular events were reported in our study population.

**Table 2:** Procedural characteristics.

Variables	*N* = 4
Technical success^a^ (*n*, %)	4 (100.0)
Number of clips (*n*, %)	
1	2 (50.0)
2	2 (50.0)
Post-procedural TR severity, grade (*n*, %)	
1+, mild	0 (0.0)
2+, moderate	3 (75.0)
3+, severe	1 (25.0)
4+, massive	0 (0.0)
5+, torrential	0 (0.0)
Reduction of ≥1 TR grade	4 (100.0)
Insertion-to-removal time (min)	93.6 ± 23.4
Fluoroscopic time (min)	30.4 ± 15.7
Procedural-related complications (*n*, %)	0 (0.0)
Mortality (*n*, %)	0 (0.0)
Myocardial infarction	0 (0.0)
Stroke (*n*, %)	0 (0.0)
Conversion to surgery (*n*, %)	0 (0.0)
Device-related reintervention (*n*, %)	0 (0.0)
Coronary artery perforation or stenosis (*n*, %)	0 (0.0)
Acute renal deterioration (*n*, %)	0 (0.0)
New conduction disturbance (*n*, %)	0 (0.0)
Access complications (*n*, %)	0 (0.0)

^a^ Technical success: Technical success was defined as: (a) the system was delivered and retrieved without occurrence; (b) at least one clip was correctly implanted and deployed as planned at the time of the patient's departure from the cardiac catheterization laboratory.

### 3.3 30-day clinical outcomes

The pre-procedure echocardiographic parameters and post-procedure clinical outcomes are presented in Table 3.

**Table 3:** Pre-procedure and 30-day echocardiographic parameters and clinical outcomes in the enrolled patients.

Echocardiographic parameters and clinical outcomes	Patient 1		Patient 2		Patient 3		Patient 4		Statistical analysis		*P*-value
	Baseline	30-day	Baseline	30-day	Baseline	30-day	Baseline	30-day	Baseline	30-day	
EROA, cm^2^	0.6	0.32	0.82	0.44	0.79	0.36	1.51	0.57	0.93 ± 0.40	0.42 ± 0.11	0.041
Rvol, ml/beat	52.0	38.0	96.5	45.0	78.5	25.0	161.0	66.0	97.00 ± 46.41	43.50 ± 17.13	0.048
Vena contracta, mm	15.0	7.6	16.0	6.7	11.0	5.6	29.8	10.0	17.95 ± 8.19	7.48 ± 1.87	0.047
Anterior-posterior annular diameter, mm	52.3	46.7	51.4	47.5	48.3	43.5	46.4	41.2	49.60 ± 2.74	44.73 ± 2.92	0.001
Septolateral annular diameter, mm	49.6	45.2	57.1	53.8	53.0	48.1	51.5	48.3	52.80 ± 3.19	48.85 ± 3.59	0.003
BNP, pg/ml	762	229	357	122	345	101	914	203	594.50 ± 287.98	163.75 ± 61.86	0.034
NYHA functional class	IV	Ⅱ	Ⅲ	Ⅱ	Ⅱ	Ι	Ⅲ	Ⅱ	–	–	–
KCCQ score	33.8	61.7	45.1	58.9	40.6	62.4	30.8	51.2	37.58 ± 6.48	58.55 ± 5.13	0.005
6MWT, m	–^a^	230	247	355	267	422	205	431	239.67 ± 31.64	402.67 ± 41.53	0.042^b^

EROA, effective regurgitant orifice area; Rvol, regurgitant volume; BNP, brain natriuretic peptide; NYHA, New York heart association; KCCQ, Kansas city cardiomyopathy questionnaire; 6MWT, 6-min walk test.

^a^ Patient 1 is completely unable to walk and cannot complete the 6-min walking test before procedure.

^b^ Patient 1's data was excluded.

**Figure 2:**
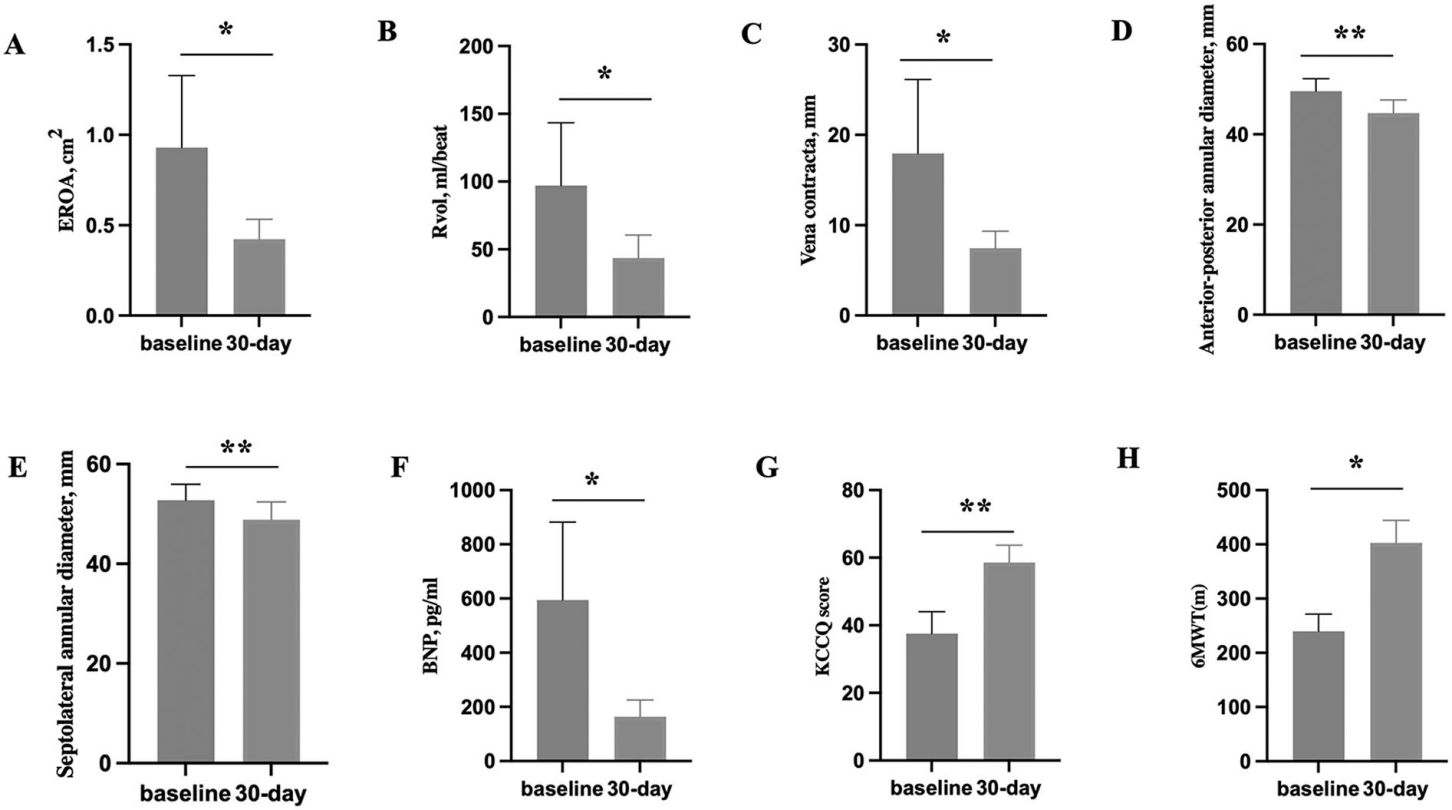
Pre-procedure echocardiographic parameters and 30-day echocardiographic parameters and clinical outcomes in the enrolled patients. **(A–C)** The comparison of tricuspid echocardiographic parameters (EROA, Rvol and vena contracta) between baseline and 30-day. **(D,E)** The comparison of the anterior-posterior annular diameter and the Septolateral annular diameter between baseline and 30-day. **(F–H)** The comparison of clinical outcomes (BNP, 6MWT and KCCQ score) between baseline and 30-day. EROA, effective regurgitant orifice area; Rvol, regurgitant volume; BNP, brain natriuretic peptide; KCCQ, 6MWT, 6-min walk test; Kansas city cardiomyopathy questionnaire; p*: *p* < 0.05, p**: *p* < 0.01.

At the 30-day follow-up visit, the mortality rate was 0, and no patients were re-hospitalized for heart failure. The vena contracta width decreased markedly from 17.95 ± 8.19 mm to 7.48 ± 1.87 mm (*p* < 0.05), as did the effective regurgitant orifice area (EROA) (0.93 ± 0.40 mm^2^ VS 0.42 ± 0.11 mm^2^, *p* < 0.05) and the total regurgitation volume (97.00 ± 46.41 ml VS 43.50 ± 17.13 ml, *p* < 0.05) (Figures 2A–C). There were obvious improvements in the NYHA functional class in 100% of patients (4 of 4), and the proportion of patients with a NYHA functional class ≥ III decreased from 75.00% (3/4) to 0. Consistent with these findings, the anterior-posterior annular diameter significantly decreased from 49.60 ± 2.74 mm to 44.73 ± 2.92 mm (*p* = 0.001), as did the Septolateral annular diameter (52.80 ± 3.19 mm vs. 48.85 ± 3.59 mm; *p* = 0.003) (Figures 2D,E).

Accordingly, the brain natriuretic peptide concentration decreased significantly from 594.50 ± 287.98 pg/ml to 163.75 ± 61.86 pg/ml (*p* < 0.05) (Figure 2F). Additionally, quality of life according to the KCCQ score improved from 37.58 ± 6.48 before the operation to 58.55 ± 5.13 at the 30-day follow-up visit (*p* < 0.01) (Figure 2G).Consistent with the improvement in the NYHA functional class, we observed an increase in the 6MWD from 239.67 ± 31.64 m to 402.67 ± 41.53 m (*p* < 0.05) (Figure 2H), in which Patient 1's data was excluded.

### 3.4 Clinical features and follow-up

Patient 1 is a 71-year-old woman presented with recurrent right heart decompensation manifesting as severe edema of the lower extremities with skin ulcerations, and chronic atrial fibrillation. She had rheumatic heart disease and mitral valve replacement 10 years ago. At the 30-day follow-up, the patient exhibited marginal enhancement in right heart function (NYHA functional class improved from IV to II) and a better overall condition, with no indications of right heart failure or edema. A notable decrease in annular diameter (10.7%) was observed, her KCCQ score increased dramatically (from 33.8 to 61.7), and the 6MWT measured 230 m (Table 3).

Patient 2 is an 81-year-old man presented with a history of severe TR. He had NYHA functional class III symptoms along with significant lower limb edema and chronic atrial fibrillation. Despite aggressive medical therapy with diuretics his symptoms persisted. At the 30-day follow-up, the patient exhibited marginal enhancement in right heart function (NYHA functional class improved from III to II) and a better overall condition, with no symptoms of right heart failure or edema. A significant reduction in annular diameter (7.6%) was observed accompanied a considerable enhancement in his KCCQ score (from 45.1 to 58.9) and 6MWT (from 247 m to 355 m) (Table 3).

Patient 3 is a 72-year-old male, with previous chronic atrial fibrillation, severe chronic obstructive pulmonary disease (COPD) and sleep apnea syndrome, presented with progressive dyspnea, lower limb edema, and palpitations in NYHA functional class II. Two years ago, he underwent percutaneous closure of the left atrial appendage due to chronic atrial fibrillation. At follow-up after 30 days, the patient showed slightly improved right heart function (NYHA functional class improved from II to I) and an improved general condition without signs for right heart failure and edemas. There was a significant reduction in annular diameter (9.9%), his KCCQ score (from 40.6 to 62.4) and 6MWT (from 267 m to 422 m) significantly improved (Table 3).

Patient 4 is a 72-year-old female, with previous chronic atrial fibrillation, severe coronary heart disease and already implanted with coronary stents, presented with progressive dyspnea, lower limb edema, and palpitations in NYHA functional class III. Anti heart failure medication can no longer alleviate her symptoms of chest tightness, wheezing, and swelling. At the 30-day follow-up, the patient exhibited a modest enhancement in right heart function (NYHA functional class improved from III to II) and a better overall state, with no indications of right heart failure or edema. A notable decrease in annular diameter (10.8%) was found, alongside a substantial enhancement in his KCCQ score (from 30.8 to 51.2) and 6MWT (from 205 m to 431 m) (Table 3).

## 4 Discussion

This report describes the early experience with the novel K-Clip™ transcatheter tricuspid annuloplasty system for TR in China. The initial experience demonstrated excellent procedural safety and good short-term efficacy. All 4 patients successfully underwent the procedure with no in-hospital or 30-day adverse events. Patients were discharged home 5–7 days after the procedure. At the 30-day follow-up visit, the tricuspid annular diameter was significantly decreased, and the NYHA functional class, distance walked during the 6MWT, and quality of life score were significantly improved.

TR can be reduced or completely eliminated with the use of surgical procedures (16). For the treatment of FTR, annuloplasty techniques—either suture-based or utilizing annuloplasty rings—should ideally be employed rather than valve replacement (16). The current recommendations (17, 18) do not include a class I indication for surgical treatment of isolated FTR. Furthermore, following standard surgery for isolated TR, comparatively significant death rates (8.8%–9.7%) have been documented (19). This could be as a result of the patients' common diseases, pulmonary hypertension and right heart failure. It is shown that all of these comorbidities indicate poorer results (20). As a result, patients might not even be referred at all or might be recommended for surgical therapy later. Several catheter-based methods that resemble surgical annuloplasty methods have been developed to solve this issue (16).

The concept of the K-Clip™ is different from that of existing TTVIs. The delivery of the K-Clip™ requires transjugular access. This access point is the same as in Kay's procedure, which is an established surgical technique for annuloplasty of the tricuspid valve (21). The Kay procedure aims to reduce TR by obliterating the annular segment corresponding to the posterior leaflet through the placement of pledged-supported mattress sutures in the annulus. As a result, the tricuspid annular circumference is reduced, and the tricuspid valve is converted into a smaller but competent mitral-like valve (21). The K-Clip™ procedure includes trans-vascular access establishment, sheath positioning, anchor deployment, annular tissue clamping, sandwich formation, and release.

Indirect annuloplasty devices for TTVIs, including the TriClip and PASCAL system, are designed to restore leaflet coaptation via edge-to-edge tricuspid valve repair through a transcatheter method, and have been reported as effective and safe for treating functional TR (22, 23). Currently, Cardioband is the first method approved for clinical use in direct annuloplasty of TTVIs devices. The Cardioband system is administered via a transfemoral technique, with the Dacron band affixed to the tricuspid annulus using a sequence of anchors positioned from the anteroseptal to the posteroseptal commissure, thereafter contracted by a size-adjustment instrument to attain annular dimension reduction. The two-year outcomes of the TRI-REPAIR research (NCT02981953) regarding Cardioband shown excellent results in individuals with symptomatic, moderate functional TR (24). However, Cardioband procedure was complicated, which limited its clinical application and promotion. K-Clip™ offers the benefits of few procedural steps, retrievability, and a sutureless design, eliminating the necessity to cross the valve or puncture the leaflets. Following K-Clip™ implantation, the tricuspid anatomy experiences minimal distortion, allowing for the continued feasibility of subsequent or contemporaneous transcatheter repair or replacement using alternative devices. Isolated tricuspid valve surgery exhibits elevated operative mortality; this transcatheter approach may present a viable alternative for addressing functional TR with satisfactory safety and simplicity.

In comparison with other techniques, the K-Clip™ procedure resulted in encouraging preliminary results in terms of safety and efficacy. The K-Clip™ procedure is rather simple, as it is not limited by the leaflet coaptation gap. However, as in other tricuspid interventions, it is highly dependent on the quality of imaging of the tricuspid valve and subvalvular apparatus, which is more challenging than imaging for mitral interventions. According to the TriStar study's data, the K-Clip™ tricuspid annuloplasty system's one-year experience led to clinical improvements in functional status and quality-of-life outcomes, as well as high survival and low rehospitalization rates with long-lasting TR reduction (25).

AF commonly occurs, especially with advancing age, and it is increasingly recognized as the leading cause of cardiovascular morbidity and mortality (26). FTR in a patient with a normal structural TV leaflet and chordae often occurs secondary to long-standing persistent AF (27). The increased prevalence of AF in the ageing population accelerates tricuspid annular dilatation and worsens TR, which in turn further dilates the tricuspid annulus and decreases RV function, therefore creating a vicious cycle of ongoing TR (8, 28). Among all existing technologies, direct annuloplasty procedures may be the most efficacious treatment for functional tricuspid regurgitation, as they directly target tricuspid annulus dilatation and may yield more enduring outcomes. Recent studies on percutaneous direct annuloplasty indicate a substantial and lasting (2 years follow-up) decrease in septo-lateral tricuspid annular diameter, with a 14% reduction in post-procedural TA diameter compared to baseline (29, 30). The one-year outcomes of the TriStar research indicated that patients who underwent the K-Clip™ operation exhibited substantial and enduring enhancements in the end-diastolic septolateral tricuspid annular diameter, with an average decrease of 11.3% (41.9 mm vs. 37.1 mm; *p* < 0.001) at one year, relative to baseline (25). A recent report reveals that tricuspid transcatheter edge-to-edge repair (T-TEER) can diminish TR by not only leaflet approximation but also reduction of TA diameter (31). In contrast to direct annuloplasty, annular reduction following T-TEER was noted in 62% of patients. Moreover, the degree of TA annular reduction observed throughout the research was comparable to that achieved with direct annuloplasty (11% for T-TEER vs. 14%) (31).

Our findings are consistent with recent observations in the published literature regarding symptomatic TR (10, 11, 25). In our study, all patients had AF, RV dilatation and systolic dysfunction with preserved LV systolic function. The predominant aetiology of TR was functional. While a TTVI is indicated when symptomatic TR is present, the risk of surgery is deemed to be too high in most patients with isolated TR due to the presence of RV dysfunction, advanced age or other (cardiac) comorbidities (17).

## 5 Conclusion

Our preliminary evaluation of transcatheter tricuspid annuloplasty with the K-Clip™ device showed exceptional procedural success and good safety outcomes for patients with severe functional TR. The severity of TR was consistently decreased through annular diameter reduction, which led to cardiac function improvement 30 days after the procedure; these outcomes were encouraging and used as the basis for larger trials to examine the safety and effectiveness of percutaneous annular diameter reduction on the prognosis in this population.

## 6 Limitations

Due to the fact that the K-Clip™ system is a pioneering tricuspid valve repair device in China, there is currently only a small amount of experience and it is still in the clinical research stage as of now. At our centre, we performed 4 such procedures to treat patients with severe TR, making the outcomes of this investigation feasible and likely not applicable to all types of patients with TR. However, our data explored the safety and effectiveness of early experience about the K-Clip™ system in china. Although the short-term clinical outcomes as shown in our study are encouraging, more data support is required for the long-term clinical efficacy. Our findings paved the way for further extensive studies to look into the safety and effectiveness of percutaneous annular reduction on the prognosis of patients with severe TR. Overall, more study on the K-Clip™ system's treatment of severe TR will benefit from bigger sample sizes and longer follow-up data.

## Data Availability

The original contributions presented in the study are included in the article/Supplementary Material, further inquiries can be directed to the corresponding authors.
